# Evaluation of the Protective Efficacy of Different Doses of a *Chlamydia abortus* Subcellular Vaccine in a Pregnant Sheep Challenge Model for Ovine Enzootic Abortion

**DOI:** 10.3390/ani14203004

**Published:** 2024-10-17

**Authors:** Morag Livingstone, Kevin Aitchison, Javier Palarea-Albaladejo, Francesca Chianini, Mara Silvia Rocchi, Sergio Gastón Caspe, Clare Underwood, Allen Flockhart, Nicholas Wheelhouse, Gary Entrican, Sean Ranjan Wattegedera, David Longbottom

**Affiliations:** 1 Moredun Research Institute, Pentlands Science Park, Bush Loan, Midlothian EH26 0PZ, UK; morag.livingstone@moredun.ac.uk (M.L.); kevin.aitchison@moredun.ac.uk (K.A.); francesca.chianini@moredun.ac.uk (F.C.); mara.rocchi@moredun.ac.uk (M.S.R.); gaston.caspe@moredun.ac.uk (S.G.C.); clare.underwood@moredun.ac.uk (C.U.); a.flockhart@napier.ac.uk (A.F.); n.wheelhouse@napier.ac.uk (N.W.); gary.entrican@roslin.ed.ac.uk (G.E.); sean.wattegedera@moredun.ac.uk (S.R.W.); 2 Biomathematics and Statistics Scotland, JCMB, The King’s Buildings, Edinburgh EH9 3FD, UK; javier.palarea@udg.edu

**Keywords:** *Chlamydia abortus*, enzootic abortion of ewes, vaccine development, vaccine efficacy, quantitative real-time polymerase chain reaction (PCR), serological analysis, cytokine analysis

## Abstract

*Chlamydia abortus* causes the disease ovine enzootic abortion, which is one of the most infectious causes of foetal death in small ruminants worldwide. While the disease can be controlled using live and inactivated commercial vaccines, there is scope for improvements in safety for both sheep and human handlers of the vaccines. We have previously reported the development of a new prototype vaccine based on a detergent-extracted outer membrane protein preparation of *C. abortus* that was determined to be more efficacious and safer than the commercial vaccines when administered in two inoculations three weeks apart. In this new study, we have developed this vaccine further by comparing its efficacy when delivered in one or two (1 × 20 µg and 2 × 10 µg) doses, as well as also comparing the effect of reducing the antigen content of the vaccine by 50% (2 × 5 µg and 1 × 10 µg). All vaccine formulations performed well in comparison to the unvaccinated challenge control group, with no significant differences observed between vaccine groups, demonstrating that the vaccine can be administered as a single inoculation and at a lower dose without compromising efficacy. Future studies should focus on further defining the optimal antigen dose to increase the commercial viability of the vaccine.

## Simple Summary

The bacterial pathogen *Chlamydia abortus* is an important infectious cause of lamb death worldwide, causing abortion in the last few weeks of pregnancy. Existing live vaccines to protect sheep from abortion are effective but have been shown to cause infection and disease in some animals. We have recently developed a new, safer prototype subcellular vaccine comprising inactivated components of the pathogen that prevent abortions occurring in sheep and cannot itself cause disease. Initial testing of this new vaccine involved administration in two shots. In this latest investigation, we have reduced this to one single vaccination that contains less antigen without affecting its effectiveness. This low-antigen single-shot delivery reduces the costs associated with manufacturing the vaccine, increasing its viability as a new commercial product.

## 1. Introduction

Ovine enzootic abortion (OEA), which is also known as enzootic abortion of ewes (EAE) and ovine chlamydiosis, is caused by the Gram-negative obligate intracellular bacterium *Chlamydia abortus* (*C. abortus*). The pathogen is one of the most common causes of infectious abortion in farm livestock in the UK [1] and worldwide [2], causing huge economic losses [3,4]. While the pathogen mainly affects sheep and goats it can also cause infection in other animal species, including cattle, pigs and horses [5,6,7,8,9]. *C. abortus* is also zoonotic and is thus a risk to immunocompromised individuals and pregnant women, in whom it can cause spontaneous abortion or result in stillbirths [8,10]. The organism poses a serious health threat to pregnant woman, causing abortion and life-threatening illness in the mother [11,12,13,14]. 

In small ruminants, the disease results in late-term abortion or stillbirths, usually within the last two to three weeks of gestation [8]. Generally, there is no advance warning of the impending event, with the first sign being the pre-term delivery of dead lambs, often in combination with the birth of weak or healthy live lambs. The placentas of infected animals are heavily infected with live infectious organisms, as are the post-parturition vaginal excretions, both of which are major sources of environmental contamination for transmission to naïve animals [8,15]. 

While both inactivated and live attenuated vaccines are available for controlling infections in sheep in Europe [16,17,18,19,20], the former have been reported to have low efficacy [21] while the latter have been associated with causing infections and disease in some animals [22,23,24,25], with genomic sequence analyses having revealed no genetic basis for any attenuation in the live vaccines [26]. Recently, we have published a study comparing the protective efficacy of two experimental subcellular vaccines prepared from sarkosyl (sodium N-lauroylsarcosine)-extracted *C. abortus* outer membrane preparations (insoluble chlamydial outer membrane protein complex (COMC) and COMC solubilised in n-octyl glucoside (OG-COMC)) in pregnant ewes experimentally challenged with *C. abortus* [27]. The COMC, which has also been shown to be protective against chlamydial diseases in guinea pigs [28] and mice [29], largely comprises the major outer membrane protein (MOMP) as its main constituent protein, comprising approximately 60% of the total outer membrane protein content [30]. Both experimental vaccines were adjuvanted with Montanide ISA 70 VG (Seppic SA, Paris, France) [31] and delivered by intramuscular (i.m.) inoculation in two doses three weeks apart. The study showed that the COMC and OG-MOMP vaccines resulted in no abortions in challenged animals, while the COMC vaccine was more efficacious than either the OG-COMC or the commercial live vaccine, resulting in a greater reduction in bacterial shedding and placental pathology [27].

The aim of this study was to further evaluate the COMC vaccine, comparing the protective efficacy of a single versus double dose, as well as to determine if the amount of protein present in each dose could be reduced by half with no adverse effect on efficacy, in order to make vaccination simpler and cheaper, thus improving its commercial potential.

## 2. Materials and Methods

### 2.1. Ethics Statement

This study was carried out in compliance with all UK Home Office Inspectorate regulations and according to the Animals (Scientific Procedures) Act 1986 and ARRIVE guidelines 2.0 [32]. The experimental protocol was approved by the Moredun Animal Welfare and Ethical Review Body (permit number: E21/13; approved on 16 April 2013). All animals were monitored at least three times daily throughout the study for any clinical signs and this monitoring increased to 24 h per day for the last four weeks of expected parturition. Any animal requiring treatment was given appropriate veterinary care (including use of antibiotics for any secondary bacterial infections) in accordance with standard veterinary practice. All lambs born weak were assessed by a registered veterinary practitioner who decided whether to euthanise the animal on welfare grounds by administrating an overdose of Pentoject^®^ (pentobarbital sodium 20% *w*/*v*; Animalcare Ltd., York, UK; #XVD133). This decision was based on different criteria, including (i) not being able to stand or lift its head and lying flat out on its side; (ii) not being able to suckle or having no interest in suckling; (iii) not being able to open its eyes; (iv) having laboured respiration; and (v) displaying minimal signs of life. All ewes and lambs were continually monitored at least three times daily following parturition for two months post-lambing and given appropriate veterinary care, where required.

### 2.2. Preparation of C. Abortus Elementary Bodies

*C. abortus* strain S26/3, isolated from the placenta of an aborted sheep that had been vaccinated with an inactivated whole-organism-based commercial vaccine [33], was propagated in McCoy cells in Corning 150 cm^2^ flasks (Scientific Laboratory Supplies Ltd., Newhouse, UK; #430825), as previously described [33,34]. Chlamydial elementary bodies (EBs) were purified on discontinuous gastrografin (Bayer, Reading, UK; #82273670) gradients, according to the method of Buendia et al. [35]. Purified EBs were suspended in 0.1 M phosphate-buffered saline (PBS), pH 7.2, and stored at −70 °C until further use. EBs were quantified using a Pierce™ BCA Protein Assay Kit (Thermo Fisher Scientific, Paisley, UK; #23227), according to the manufacturer’s instructions, following solubilisation of the antigen in 0.2 M sodium hydroxide for 1 h at 37 °C.

### 2.3. COMC Preparation, Quantification and Formulation into Experimental Vaccines

COMCs were prepared from stored EBs for formulation into the experimental vaccine and for use in the cellular studies by sequential detergent extraction in 2% sarkosyl (Merck Life Science UK Ltd. (Sigma-Aldrich Co.), Gillingham, UK; #61743), followed by 2% sarkosyl containing 10 mM dithiothreitol (DTT; Promega, Southampton, UK; #V3151) and differential centrifugation, as described previously [27,36]. The final insoluble COMC pellet was resuspended in PBS, quantified by densitometry of the MOMP band on an SDS-PAGE gel, as previously described [27], and stored at −70 °C until formulated into the vaccine. The COMC is identical to that reported previously in a publication providing a detailed analysis of the protein constituents of the preparation [30].

COMCs were diluted in PBS to prepare the antigenic aqueous phase of the vaccine prior to adjuvanting with Montanide™ ISA 70 VG (Seppic SA) [31], according to the manufacturer’s instructions, using a ratio of adjuvant/antigen of 70/30 (weight/weight). Three experimental vaccine formulations were prepared containing final concentrations of 20 µg, 10 µg and 5 µg equivalent MOMP per 1 mL dose. A stable emulsion was achieved using an Ultra Turrax homogeniser (IKA^®^-Werke GmbH & Co., Staufen im Breisgau, Germany) at high shear rate at room temperature. Vaccines were prepared and stored at 4°C for one month prior to administration to ensure stability.

### 2.4. Preparation of C. Abortus Challenge Inoculum

Challenge inoculum was prepared from *C. abortus* strain S26/3 in fertile hens’ eggs, as previously described [37,38], and challenge material was stored in liquid nitrogen until required. The titre of the *C. abortus* yolk sac material was determined following inoculation of McCoy cell monolayers grown on coverslips, growing at 37 °C under CO_2_ for 72 h and counting the number of infected cells [27]. Immediately before challenge, the inoculum was removed from liquid nitrogen storage and diluted in PBS to 10^6^ inclusion-forming units (IFUs) of *C. abortus* per 1 mL.

### 2.5. Experimental Design

**Figure 1:**
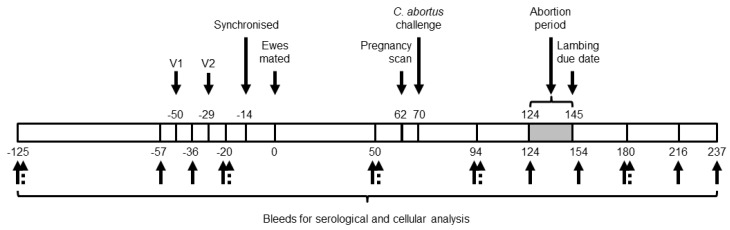
Experimental design. Numbers above and below the bar indicate the number of days prior to or post-mating. Animals received one (V1) or two (V1 and V2) vaccine doses at seven (V1) and four (V2) weeks prior to mating. Bleed dates (weeks prior to and post-mating) for serological (solid arrows) and cellular (dotted arrows) analysis are as indicated.

Sheep (Scotch Mules which are crossbred Scottish Blackface ewes sired by Bluefaced Leicester rams; aged 1 to 3 years; n = 233) were obtained from our regular source flock and pre-screened, as described previously [27], by rOMP90-3 enzyme-linked immunosorbent assay (ELISA) and in vitro lymphocyte stimulation assay to check for any prior exposure to *C. abortus*. A total of 150 animals were selected for the study and assigned randomly to 6 groups, each containing 25 ewes. Groups 1 to 4 were vaccinated as follows: group 1 animals were vaccinated twice each with 1 mL of the COMC vaccine containing 10 µg of MOMP per 1 mL dose at seven and four weeks prior to mating; group 2 animals received a single 1 mL dose of the COMC vaccine containing 20 µg of MOMP per 1 mL at seven weeks before mating; group 3 animals were vaccinated twice each with a 1 mL dose of the COMC vaccine containing 5 µg of MOMP per 1 mL at seven and four weeks prior to mating; and group 4 animals received a single 1 mL dose of the COMC vaccine containing 10 µg of MOMP per 1 mL at seven weeks before mating. All primary vaccinations were administered intramuscularly (i.m.) using a 19G one-inch needle on the left side of the neck, while secondary vaccinations in groups 1 and 3 were administered i.m. on the right side of the neck. Group 5 and 6 animals were not vaccinated and served as positive and negative controls, respectively. Three weeks after secondary vaccinations, all ewes were synchronised for oestrus using Chronogest^®^ CR 20 mg controlled-release sponges (MSD Animal Health UK Ltd., Milton Keynes, UK) over two weeks and then mated. At day 70 of gestation, all pregnant vaccinated ewes (groups 1-4) and group 5 positive control ewes were inoculated subcutaneously (s.c.), using a 19G one-inch needle, over the left prefemoral lymph node with 2 mL of challenge inoculum containing 2 × 10^6^ IFU of *C. abortus* strain S26/3. Group 6 animals served as unvaccinated and non-challenged negative controls and were housed separately from the other groups. All animals were fed on a normal diet with free access to hay and water. The clinical outcome of each ewe was recorded, as well as the weight and sex of each lamb/foetus immediately after delivery. A summary of the experimental design is depicted in Figure 1. 

### 2.6. Sample Collection and Analyses

Placentas were recovered at lambing or abortion, examined for evidence of typical OEA lesions [23,37,39,40,41] and analysed for the presence of *C. abortus*, as previously described [27]. The extent of gross pathology was estimated as a percentage of the area affected. Multiple samples of cotyledons plus the surrounding intercotyledonary membrane were excised from each placenta and placed into CellPath specimen containers pre-filled with 10% neutral buffered formalin (BF) (Fisher Scientific UK Ltd., Loughborough, UK; #13191184) for routine histological examination and immunohistochemistry (IHC) to confirm OEA. For any foetuses recovered from a suspected case of atypical abortion, samples of brain, lung, heart and liver were placed in 10% BF for histopathological investigation and IHC. These organs are routinely sampled in pathology laboratories to confirm chlamydial infection (liver), as well as discount other common causes of ovine fetopathy in the UK, including protozoa such as *Toxoplasma gondii* (brain and heart) and bacterial pathogens *Campylobacter* and *Salmonella* spp. (liver and lung). Lung and brain samples are also useful for confirming suffocation resulting from dystocia.

Samples of placental cotyledons were excised and smears prepared for modified Ziehl–Neelsen (mZN) staining [42] to confirm the presence of chlamydial organisms, as previously described [27]. Genomic DNA was extracted from approximately 25 mg of placental cotyledon tissue samples using a DNeasy^®^ Blood and Tissue Kit (Qiagen Ltd., Crawley, UK; #69504), as previously described [27]. Extracted DNA was tested in triplicate wells by *C. abortus* OmpA quantitative real-time polymerase chain reaction (qPCR) [27,41] and results were expressed as the number of *C. abortus* genome copies per 2 µL total placental-extracted DNA to estimate chlamydial load. 

Blood samples (10 mL) were collected into BD Vacutainer^®^ serum tubes (Fisher Scientific UK Ltd., #12957686) prior to vaccination and at regular intervals throughout the study for serological analysis by ELISA, while additional 10 mL blood samples were collected in BD Vacutainer^®^ heparin tubes (Fisher Scientific UK Ltd., #13171543) for use in cellular assays (interferon-gamma (IFN-γ) production only) (Figure 1).

### 2.7. Histopathological Examination and Immunohistochemical Analysis

Placental and foetal tissues were fixed in 10% BF for 4–10 days, trimmed, dehydrated through graded alcohols, cleared in xylene and embedded in paraffin wax using standard protocols. Prior to histopathological and IHC analysis, 5 μm serial sections were cut and stained with haematoxylin and eosin for histopathological examination. Sections were labelled for IHC with a mouse monoclonal antibody (mAb) to the lipopolysaccharide (LPS) of *C. abortus* strain S26/3 (mAb 13/4; Santa Cruz Biotechnology, Inc., Heidelberg, Germany; #sc-101593), visualised using a goat anti-mouse IgG conjugate (Dako EnVision™+ System HRP-labelled polymer (Mouse); Agilent Technologies Denmark ApS, Glostrup, Denmark; #K4001), counterstained with haematoxylin and mounted, as previously described [23]. 

### 2.8. Serological and Cellular Interferon-Gamma Analyses

Antibodies to *C. abortus* were measured by analysing serum samples, which were collected from all animals throughout the duration of the study, by rOMP90B-3 ELISA, as previously described [43]. Results were expressed as percentage values following normalisation of optical densities using positive and negative control sera using the following formula: [(OD sample − OD negative control)/(OD positive control − OD negative control)] × 100, as previously described [43]. 

Ovine peripheral blood mononuclear cells (PBMCs) were isolated from venous blood (10 mL/sheep collected into heparinised tubes), counted, adjusted to 2 × 10^6^ cells/mL in complete Iscove’s Modified Dulbecco’s Medium (IMDM) supplemented with 10% heat-inactivated FBS, 2 mM L-glutamine, 50 µg/mL gentamycin, 50 µg/mL streptomycin, 50 µM beta-mercaptoethanol and 100 IU/mL penicillin and cultured in 96-well U-bottom plates (Nunc) for 96 h, according to previously described protocols [44]. Antigen-specific recall responses were assessed by analysing culture supernatants from quadruplicate wells collected 96 h after stimulation with 100 μL of purified *C. abortus* EBs (5 μg/mL), ConA (5 μg/mL; concanavalin A from *Canavalia ensiformis*, MP Biomedicals, Fisher Scientific UK Ltd.) or medium alone, as described previously [45]. For IFN-γ measurement, a standard sandwich ELISA protocol was followed [46] using the species cross-reactive bovine IFN-γ mAb clones CC330 and CC302b (Bio-Rad Laboratories Ltd., Hertfordshire, UK) [47] and quantification was undertaken using recombinant bovine IFN-γ (Endogen-Pierce Biotechnology, Rockford, IL, USA). 

### 2.9. Statistical Analyses

Data on abortion incidence were modelled using a generalised linear model (GLM) assuming a binomial distribution and a logit link function. Model parameters were estimated by the maximum likelihood method, including a bias-reduction correction [48] to accommodate lack of variability in outcome within some treatment groups. The models included the group as the explanatory variable and the overall statistical significance of the group effect was assessed based on the chi-square statistic.

PCR quantification data were summarised using geometric mean and geometric standard error of the mean (SEM) in accordance with the characteristics of their distribution (highly right-skewed positive values; geometric statistics obtained from ordinary statistics computed on (log + 1)-transformed data and exponentiated to be expressed in original units). Placental qPCR loads were statistically compared between treatment groups by non-parametric rank-based testing, using Dunnett’s contrasts to compare vaccinated groups with the challenge control group and Tukey’s contrasts for comparisons amongst vaccinated groups [49].

Serological (ELISA) responses at different time points were compared between groups using a linear mixed model (LMM) with identity link function and Gaussian errors fitted by restricted maximum likelihood to rank-based inverse normal transformed data. Group, time (day of gestation) and a group/time interaction term were included in the model as fixed-effect factors, whereas animal ID was specified as a random effect. An analogous LMM was formulated to compare cytokine IFN-γ responses at different cellular bleeds, including group, stimulant and their interaction as fixed effects. Significance of the fixed effects was assessed by conditional F-tests. Post hoc pair-wise comparisons used t-tests based on marginal means estimated from the LMM fits.

All statistical analyses were carried out on the R system for statistical computing v4 [50], with statistical significance assessed at the usual 5% significance level. Where multiple comparisons of groups were conducted, p-values were adjusted to control for false discovery rate (FDR) using the Benjamini–Hochberg method [51].

## 3. Results

### 3.1. Clinical Outcome of Pregnancy

The pregnancy rates following mating and pregnancy outcomes for each of the four vaccinated (groups 1–4) and two control groups (groups 5 and 6) are shown in Table 1. For the purposes of the results and statistical analyses, pregnant ewes were considered to have aborted if they delivered at least one dead foetus or a live non-viable (weakly) lamb that had to be euthanised on animal welfare grounds or one which died within 48 h of birth (i.e., neonatal deaths and stillbirths). Abortions were classified as due to *C. abortus* if chlamydial organisms and/or DNA could be detected by mZN, qPCR or following pathological investigations of placentas, foetuses or uterine discharges post-parturition. 

There were no abortions from any of the negative control ewes (group 6) with all animals lambing at the expected time (gestational range of 139–149 days; Table S1). In challenge control group 5, seven of the twenty-five pregnant ewes aborted a total of six foetuses and gave birth to five weakly lambs that were euthanised on humane grounds or died shortly after birth (four lots of twins and three individuals) and had a gestational range of 121–141 days (the range was 133–149 days for those ewes that delivered live lambs; Table S1). All ewes in the experimental vaccine groups delivered healthy live lambs (range of 139–151 days), apart from a single ewe in group 3 that delivered two dead lambs two weeks prior to expected parturition (day 132 of gestation; Table S1). Although there was a single abortion event in the vaccinated group 3 ewes, there was a statistically significant lower abortion rate in this group (and in groups 1, 2 and 4) when compared to the positive control (group 5) animals (*p* = 0.0194).

### 3.2. Detection of C. Abortus Infection

Assessment of all the placentas collected from the vaccinated animals in groups 1–4 that gave birth to apparently healthy lambs revealed no evidence of any gross pathology (Table 2). The ewe in group 3 that aborted twins delivered two placentas, both of which had clear evidence of placental pathology covering the entire placental surface. Similar high levels of gross placental pathology were observed for the lambs that aborted in the challenge control group 5 (varied between 40 and 100%). However, in this group, placental gross pathology was also observed for two of the ewes that gave birth to apparently normal lambs, although this pathology was less extensive (5–40%) than for placentas for the aborted lambs (see complete data for individual animals in Table S1).

The analysis of placental smears to identify the presence of organisms following mZN staining completely matched the observed gross pathology results, including organisms being detected in the placentas of the apparently healthy lambs with evidence of gross pathology that were delivered from the two challenge control ewes (Table 2). Similarly, placental smear mZN scores were higher for the placentas from aborted lambs than for those that survived, again matching the observed difference in the level of gross placental pathology (Table S1). 

Placental qPCR analysis added an additional level of sensitivity, above the more qualitative gross placental pathology and placental smear observations, such that groups 2–5 each had an apparent increase in the number of ewes that lambed for which organisms could be detected above the threshold level of 100 genome copies in the placentas. This was much more evident for the group 5 challenged ewes where 17/18 animals that lambed had placental qPCR-positive samples greater than 100 genome copies. Thus, when considering the presence of placental gross pathology, organism and organism DNA (herein referred to as the “infection rate”), this infection rate was statistically significantly higher than for the vaccinated groups (*p* < 0.0001). These group 5 challenge control placentas also had a much higher organism load detected when compared to similar samples from the vaccinated groups 1–4, as indicated by the geometric means shown in Table 2 (1481 versus 2–35 *C. abortus* genome copies). In contrast, qPCR of placentas from the single aborted animal in vaccinated group 3 and the seven that aborted in challenge control group 5 revealed much higher organism loads (note the large geometric means in Table 2).

Thus, overall, the mean number of organisms detected in placentas was considerably lower for the vaccinated groups, reflecting the skew of the data towards lower values for most of the animals (Table S1). Indeed, while 24/25 animals had organism loads ranging from 2.9 × 10^2^ to 9.35 × 10^7^ genome copies in group 5 (with the remaining one animal below 36), all the animals in groups 1 and 2 (group 1, range of 0 to 69; group 2, range of 0 to 164), 20/23 animals in group 3 (range of 0 to 107; remaining three animals, 1.8 × 10^3^, 12.2 × 10^3^ and 1.95 × 10^6^) and 21/22 animals in group 4 (range of 0 to 183; remaining one animal 6.6 × 10^2^) were below this range. When considering the four vaccinated groups, there was a statistically significant reduction in organism load when compared to the challenge control group (*p* < 0.0001). Within the vaccinated groups, no statistically significant differences were identified between groups 1, 3 and 4 (*p* > 0.1499), which overall appeared to have the greatest reduction in placental infection. A significantly higher placental qPCR load was statistically detected in group 2 when compared with the other vaccinated groups (*p* < 0.0001); however, this did not lead to significantly different infection rates amongst them (*p* > 0.1183). No evidence of infection in any of the placentas from the negative control group 6 was observed, as would be expected.

### 3.3. Histopathology and Immunohistochemical Analyses

Histological and IHC analysis was performed on a random selection of placentas and foetuses and where an unusual event occurred, such as an atypical abortion or when a lamb was found dead and wrapped in its membranes. In keeping with the clinical outcome, the analysed samples were observed to exhibit different degrees of pathology. This was particularly evident in the challenge control group 5 and the single abortion in group 3 where suppurative necrotising placentitis with vasculitis was observed in the affected placentas. This, along with positive labelling by IHC, was diagnostic of *C. abortus* infection and was indistinguishable from pathology previously reported for this disease (not shown) [23,41,52]. Two lambs were found dead, wrapped in their placental membranes (one each in groups 1 and 3; see Section 3.1); however, histology and IHC revealed no lesions or antigen labelling typically associated with OEA in any of the tissue samples examined. No evidence of pathology in any of the negative control animals or placentas examined was observed. 

### 3.4. Serological and Cytokine Interferon-Gamma Pre-Screening

Pre-screening of 234 animals, sourced from a flock routinely used for our *C. abortus* sheep studies, by serological ELISA revealed unusually high numbers of animals to be positive (n = 84; 36%), although no abortions were reported in the flock. The remaining seronegative animals (n = 150) were re-screened and confirmed as seronegative prior to vaccination and mating. A single set of cellular recall assays was performed as part of the pre-screening to assess background responses to unstimulated PBMCs, antigen-specific responses to *C. abortus* EBs and the capacity of cells to respond to ConA (based on the screening approach previously undertaken [45]). These assays revealed that most of the responses to unstimulated cells were low/negligible, whereas, as expected, they were high to the T cell mitogen ConA. Analysis of the antigen-driven responses revealed that most sheep had a high level of pre-existing cellular immunity to the *C. abortus* antigen with levels of individual sheep often exceeding values from ConA treatment samples. Taken together with the serological data, this is suggestive of prior exposure to *C. abortus*. The recall antigen preparation used here included whole *C. abortus* EBs and therefore will likely show immune reactivity resulting from exposure to other chlamydial pathogens, such as *Chlamydia pecorum* and/or *Chlamydia psittaci*. Due to time constraints regarding the trial schedule and the difficulty in obtaining and screening another batch of animals, not to mention the associated costs, an institutional decision was made following discussion with the Chairperson of the Moredun Animal Welfare and Ethical Review Body to proceed with the trial as planned using the serologically negative animals. 

### 3.5. Serological Responses

**Figure 2:**
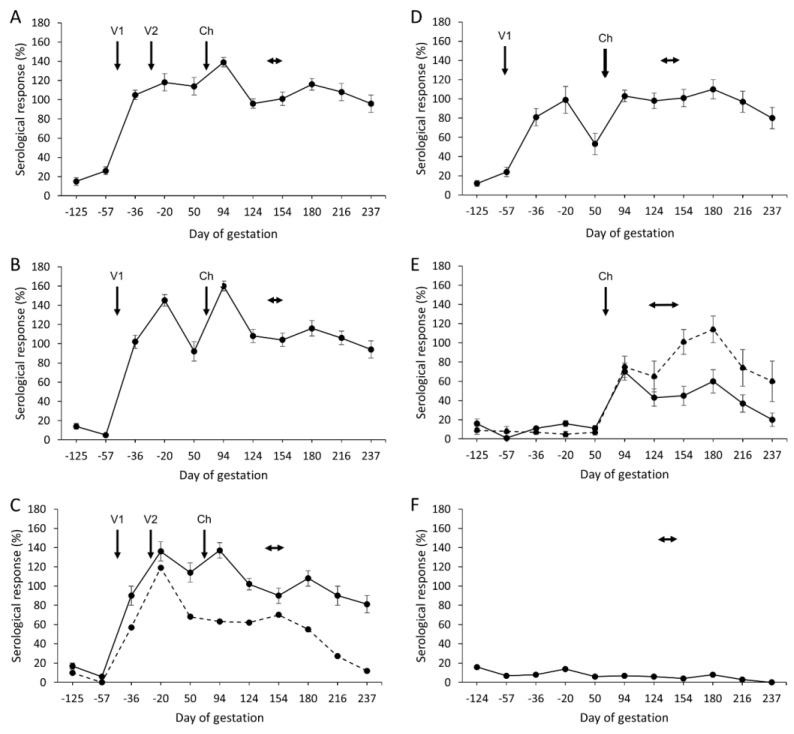
Serological responses following vaccination and challenge with *C. abortus*. Detection of *C. abortus* antibody in ewes vaccinated with one (V1) or two (V1 and V2) doses of the experimental COMC antigen preparation (2 × 10 μg (**A**), 20 μg (**B**), 2 × 5 μg (**C**) and 10 μg (**D**)) and challenged (Ch) on day 70 of gestation with *C. abortus* strain S26/3. Unvaccinated challenged (**E**) and unvaccinated non-challenged (**F**) ewes served as positive and negative control groups. Data are separated into lambed (solid lines) versus aborted (dotted lines). Data points represent the arithmetic mean values for each cellular bleed and error bars represent the standard error of that mean (SEM). A value of 100% is equivalent to an OD450nm of 2.25. The lambing/abortion period for each group is indicated by the horizontal double-headed arrows.

The mean antibody responses for the aborted and lambed animals in each of the vaccinated challenged groups and control groups are shown in Figure 2. A clear antibody response was elicited following vaccination with all four experimental vaccines (Figure 2A–D), being statistically significantly higher than the mean response observed for the challenge and negative control groups (Figure 2E,F, *p* < 0.0001). There were no statistically significant differences in the magnitude of the mean responses regardless of receiving two or single doses of antigen (*p* > 0.0678), with the exception of the single 10 µg dose group (Figure 2D; group 4), where the response appeared to be significantly lower than when using the single 20 μg dose (*p* = 0.0247; Figure 2B; group 2). The antibody responses in the vaccinated groups waned just prior to challenge, although not by much, in the group receiving 2 × 10 µg COMC (Figure 2A), but following challenge at day 70 of gestation, the responses transiently increased in magnitude and then declined both pre- and post-parturition. It was noted that for the single ewe that aborted in group 3 (Figure 2C), there was no transient increase in the magnitude of the response after challenge, and following parturition, the response rapidly declined.

Reassuringly, the challenge control animals (group 5) remained seronegative prior to administration of the challenge, following which there was an increase in antibody response, which then dipped in magnitude and following parturition rapidly decreased (Figure 2E). Although the mean response was greater in the animals that aborted in this group than in those that lambed from around 94 days of gestation onwards, there was no statistically significant difference between lambed and aborted animals when the entire timespan was considered (*p* = 0.1991). This reflects the fact that all of the animals in this group except one showed strong evidence of heavy infection in the placentas in terms of gross pathology, mZN staining of placental smears or qPCR detection of organisms in placental extracts, irrespective of pregnancy outcome.

All the animals in negative control group 6 (Figure 2F) remained serologically negative throughout the experiment.

### 3.6. Cellular Interferon-Gamma Responses

**Figure 3:**
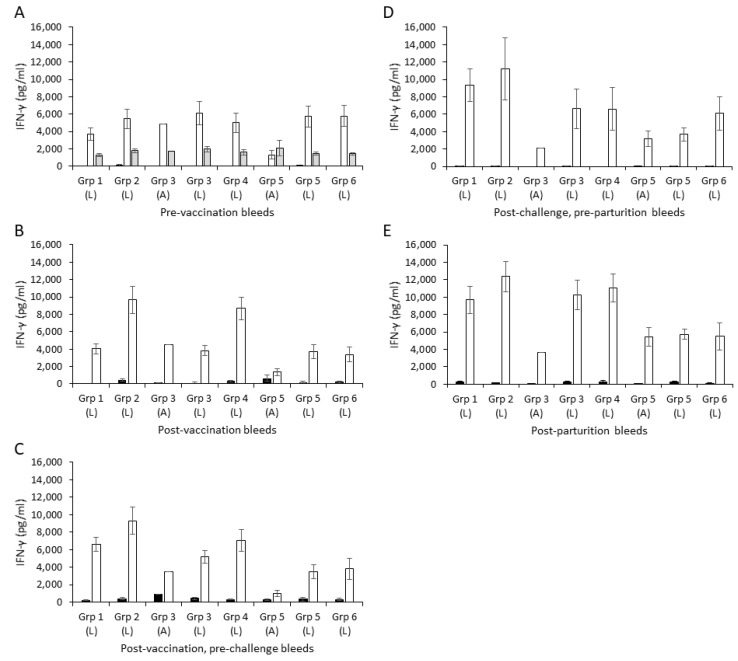
Interferon-γ responses following vaccination and challenge with *C. abortus*. Peripheral blood mononuclear cells (PBMCs) from the vaccinated challenge control and negative control groups were purified from whole blood (as described in Materials and Methods) collected pre-vaccination (**A**), post-vaccination (**B**), post-vaccination/pre-challenge (**C**), post-challenge/pre-parturition (**D**) and post-parturition (**E**) (also see Figure 1). PBMCs were set up in lymphocyte stimulation assays in vitro using medium only as an unstimulated cell control (black bars), the mitogen Concanavalin A (ConA) as a positive control (panel A only; grey bars) and UV-inactivated *C. abortus* EB antigen (no fill bars) to measure chlamydial antigen-specific stimulation. Antigen-specific IFN-γ recall responses were assessed by analysis of the culture supernatants. Data points represent the mean values for each cellular bleed and error bars represent the standard error of that mean (SEM). L, lambed ewes; A, aborted ewes.

The baseline pre-vaccination antigen-specific cellular responses of the cohort of sheep were consistently higher than those observed with unstimulated cells (*p* < 0.001), particularly the *C. abortus* EB antigen responses, and were broadly consistent across the groups other than for the aborted animals in the challenge control group (*p* = 0.8351) (Figure 3A). ConA responses are usually provided as a positive control in cellular assays to demonstrate cells are live and capable of responding to polyclonal stimulation. In this study, given the high responsiveness of PBMCs to EB antigens, the ConA treatment was analysed in the pre-vaccination bleed only (Figure 3A) to demonstrate consistency in the capacity of PBMCs across groups to respond to mitogenic stimulation. The IFN-γ response to medium alone remained consistently low throughout the groups for all bleeds. In this study, and to our surprise, pre-vaccination chlamydial EB antigen responses were between 3000 and 5000 pg/mL, which was remarkably high in comparison to what we have observed in previous studies [27,45].

In contrast to the pre-vaccination bleed, differences were observed in the magnitude and kinetics of the antigen-driven IFN-γ responses of the animals in the different vaccine groups (1 to 4) to vaccination. In the post-vaccination, pre-challenge samples (Figure 3B), there was selective elevation of antigen-driven recall responses in the single-vaccine inoculation groups 2 (*p* < 0.0020) and 4 (*p* < 0.0060) above baseline that were maintained at this level in the following pre-challenge bleed (Figure 3C; *p* = 0.6870 for group 2 and *p* = 0.1331 for group 4). No statistically significant differences were detected between groups 2 and 4 at any of these bleeds (*p* > 0.4403). For groups 1 and 3, which received two inoculations, lower responses were generally observed than in groups 2 and 4 (*p* < 0.0053). Specifically, in group 1, a delayed elevation was observed in bleed 3 (Figure 3C versus 3B), while there was no elevation observed for the group 3 aborted or lambed animals (Figure 3B,C versus 3A). The challenge control group 5 and negative control group 6 responses remained consistently between 4000 and 6000 pg/mL in all pre-challenge bleeds. Following experimental chlamydial challenge, elevated responses were observed across groups (in post-challenge/pre-parturition bleeds (Figure 3D) and in post-parturition bleeds (Figure 3E), apart from for the aborted animal in group 3. 

## 4. Discussion

In a previous study [27], we compared and evaluated two experimental subcellular vaccine preparations based on detergent-extracted fractions of *C. abortus* EBs (COMC and OG-COMC), which were delivered in two inoculations (2 × 10 μg doses), while an additional group of animals were inoculated with one of the live attenuated commercial vaccines (Cevac^®^ Chlamydia, Ceva Animal Health Ltd., Amersham, UK). That study found the COMC vaccine to be the most efficacious in terms of achieving the lowest abortion and organism shedding rates at parturition. In the present study, we evaluated the COMC vaccine further to determine whether we could achieve the same level of efficacy by delivering the vaccine in a single dose rather than two, as well as determining whether we could halve the antigen dose administered without adversely affecting efficacy. The evaluation was conducted using our well-established pregnant sheep challenge model [37,41] and using the same Montanide^TM^ ISA 70 VG adjuvant that we used in the previous study [27]. We have shown in many similar studies over the last 34+ years that this pregnant sheep model is highly reproducible and reliable in mimicking a natural infection in terms of placental pathology and immunological responses [16,27,36,37,41,45] and thus is the best validated natural model for assessing the protective efficacy of experimental *C. abortus* vaccines.

Prior to commencing the study, we initially pre-screened 200 animals, which were sourced from a flock routinely used for our *C. abortus* sheep studies, by serological and cellular IFN-γ analyses. However, these analyses showed unusually high numbers of animals to be positive by both assays. The animals showed a high level of pre-existing cellular immunity to the *C. abortus* antigen, suggestive of prior exposure to the pathogen or perhaps another related chlamydial pathogen, *C. pecorum*, which is commonly found in sheep [53,54]. These results came as a surprise as no abortions had been reported in the flock that the animals were obtained from and there had been no previous exposure to the pathogen reported in this flock. Following discussion with the Moredun Animal Welfare and Ethical Review Body and on the basis that we had enough serologically negative animals to proceed, there was agreement to continue with the trial. At this stage, the selected animals were re-screened and still found to be serologically negative. This gave us some reassurance that the animals were truly negative, which was further reinforced by subsequent low serological screening results obtained prior to vaccination/challenge in the vaccinated groups and the challenge control group, as well as throughout the study in the negative control group. Nonetheless, we have taken all this into consideration when interpreting the results we obtained in this study. It should also be noted that no abortions were subsequently reported in the flock that we obtained the animals from in the following lambing season. 

When only considering clinical outcome for evaluating vaccine efficacy, we found that three of the vaccinated groups resulted in no abortions. One abortion event was recorded in the group receiving two doses of 5 μg COMC. Despite this single abortion, there was still a clear statistically significant difference between all the vaccine groups compared to the challenge control group. But could this lack of abortion in vaccinated animals be due to possible pre-exposure to the pathogen within the flock, as discussed above? If this had been the case, then we would expect the same thing to occur in the challenge control group. Therefore, the fact that abortions (28% abortion rate) occurred in the challenge control group suggests that this is not a likely possibility or that not all sheep within the groups had prior exposure to *Chlamydiae*. Indeed, there is also an argument that vaccinating animals over an existing infection might mean the vaccine is not able to overcome prior exposure, which could result in more abortions occurring [55]. So overall, taking all these points into consideration, we can conclude that the vaccinations were effective at reducing abortion in the challenged animals.

We also considered other factors in addition to the clinical outcome in order to fully assess the efficacy of the different vaccine regimens. These factors, which have an important role in the transmission of infectious organisms following parturition to susceptible naïve animals, specifically relate to the products of abortion, namely the placentas and dead foetuses [8]. Therefore, we also evaluated the extent of gross pathology and the presence (by mZN) and load (by qPCR) of organisms in the placentas, as measures of infection and as indicators of potential contributory factors for transmission. The placental qPCR data showed an increase in sensitivity for identifying organisms compared to the more qualitative assessments of estimating gross placental lesions and organism presence/load in placental smears, both of which showed complete agreement. This was particularly evident with the lambed animals in the challenge control group where 17 of 18 animals were deemed to show evidence of infection, compared to only two following placental lesion and smear scoring. Perhaps not surprisingly, the qPCR results for these 17 animals were considerably lower (range, 2.9 × 10^2^ to 6.8 × 10^6^ *C. abortus* genome copies; geometric mean, 1.5 × 10^3^ copies) than those obtained for the aborted animals (range, 2.2 × 10^4^ to 9.3 × 10^7^ *C. abortus* genome copies; geometric mean, 8.2 × 10^6^ copies), reflecting the fact that they lambed normally despite the relatively high load of pathogen present in the placentas. A total of eight of the lambed animals within three of the vaccinated groups (three each in groups 2 and 3; two in group 4) were also deemed positive by placental qPCR only. However, the results were considerably lower than those observed for the challenge control animals that lambed, as reflected by the lower geometric means (34.7, 2.80 and 5.25 for groups 2–4, respectively, versus 1481 for group 5). Specifically, five of the eight animals had qPCR results that were not significantly higher than the 100 genome copy cut-off (range of 107 to 183 copies), while the remaining three, although higher (group 3, 1.77 × 10^3^ and 1.22 × 10^4^ copies; group 4, 660 copies), were still considered relatively low compared to what was observed in the challenge control animals. So overall, although there was evidence of infection in these lambed animals, the infection was considered to be low, giving us confidence that the vaccines had sufficiently reduced organism burden, whether administered as two (2 × 10 μg and 2 × 5 μg antigen) or single (20 μg and 10 μg antigen) doses, and thus can be considered as effective at reducing infection/abortion and resulting in a successful lambing. Additionally, the low level of infection in the placentas suggests a low potential risk of transmission of infection to other naïve animals. 

A single abortion event occurred in group 3 that resulted in two dead lambs, the placentas of which had a very high pathogen load, as estimated by qPCR (geometric mean of 1.7 × 10^6^ copies), comparable to that observed in the aborted challenge control group (group 5). Although the animal responded serologically to the two 5 µg doses of vaccine administered, there was no obvious increase in cellular IFN-γ. The antibody response then rapidly declined and there was no antibody or IFN-γ response to the challenge observed. While it could be argued that the lack of antibody response could be linked to the establishment of infection and abortion occurring, this is not what we have previously observed [27,37,41] in aborted animals, where the antibody response is generally greater in aborted animals due to the placental infection caused by the challenge, resulting in greater antigenic stimulation. Indeed, an investigation of the placentas revealed pathology typically associated with OEA, suggesting the abortion was due to the administered challenge, while no other abortifacient agents were detected. So why the vaccine appeared to have had no effect in priming the immune system to the challenge in this particular animal remains unclear. 

Antibody responses were elevated following vaccination in all four experimental groups, regardless of whether they received one or two doses of antigen. While there was a lower antibody response observed for the animals receiving a single 10 μg dose (group 4), this difference was not statistically significantly different to the other three vaccinated groups. In general, the four vaccinated groups showed very similar antibody profiles throughout the entire length of the study, with the exception of a bigger drop in antibody response observed pre-challenge in the groups receiving a single dose of antigen. These observed antibody profiles were very similar to those observed previously [27]. This includes the greater antibody response observed in the aborted animals compared to those that lambed in the challenge control group 5, which, as we have discussed in our previous study [27], supports the view that antibody has little or no role in protecting the establishment of placental infection that ultimately leads to abortion [56,57]. This also fits with the importance of a cellular response, rather than humoral response, and particularly IFN-γ being important in controlling *C. abortus* infections [58,59]. Therefore, we also evaluated antigen-specific IFN-γ production for each of the groups in this study. As we have noted earlier, the chlamydial EB antigen-specific responses were found to be higher across all the groups than what we have observed in our previous studies [27,37,45], which suggests pre-exposure to the pathogen. Despite this, post-vaccination and pre-challenge bleeds were generally elevated above the baseline levels, particularly in the single-vaccine inoculation groups 2 and 4, regardless of dose received. While the responses were generally lower pre-challenge in groups 1 and 3 receiving two vaccine inoculations, the post-challenge and post-parturition responses were elevated in all ewes that lambed in the four vaccinated groups. The only exception was again the ewe that aborted in group 3 where responses did not differ at each sampled time point, which suggests no enhanced capacity to respond to *Chlamydiae* following vaccination. Therefore, overall, the data indicate an association of higher responses in vaccinated sheep that have lambed outcomes, although with the caveat that there is only a single abortion event in the vaccinated groups. However, the differences are marginal and not found to be statistically significant and nonetheless suggest that there is an effect of the number of inoculations and the vaccine dose on the sheep cohort despite possible pre-exposure to *C. abortus*.

## 5. Conclusions

In this study, we have further evaluated a new prototype vaccine for controlling *Chlamydia abortus* infections in sheep. Although there was evidence of pre-exposure to the pathogen in the flock from which the sourced animals were derived, all subsequent analyses show, at least serologically and pathologically, the selected screened animals used in the study were free of infection up to the point of administering the challenge. The results of the study show that the vaccine retains good efficacy when delivered in a single inoculation rather than two and that halving the dose has no detrimental effect on this protective efficacy. The vaccine warrants further evaluation to investigate whether the antigen dose can be reduced further in a dose response-type experiment to determine the lowest permissible dose while not compromising efficacy. This will help in keeping production costs at a reasonable price to ensure its potential future commercial viability. Thus, this study has demonstrated that the novel single-shot subunit vaccine for *C. abortus* is safe, immunogenic and protective, with the potential for further refinement of antigen content, all of which are competitive qualities compared to the existing commercial vaccines.

## Figures and Tables

**Table 1:** The clinical outcome of pregnancy in vaccinated ewes that were subsequently challenged with *Chlamydia abortus* at day 70 of gestation (groups 1–4), in infected control ewes (group 5) or in uninfected control ewes (group 6).

Group	Ewes				Number of Lambs		
	No. Pregnant	No. Lambed (%)	No. Aborted (%)	Mean Gestational Length	Viable	Non-Viable	Dead
1	24	24 (100)	0	145	43		1 ^1^
2	25	25 (100)	0	144	39		0
3	23	22 (96)	1 (4)	145	35		3 ^1^
4	22	22 (100)	0	145	32		0
5	25	18 (72)	7 (28)	141	33	5 ^2^	6
6	23	23 (100)	0	145	37		0

^1^ includes death of one lamb in each of groups 1 and 3 due to dystocia/asphyxiation. ^2^ neonatal deaths (born live but died within 48 h). Group 1, 2 × 10 μg COMC; group 2, 1 × 20 μg COMC; group 3, 2 × 5 μg COMC; and group 4, 1 × 10 μg COMC. Group 5, challenge controls. Group 6, negative controls.

**Table 2:** Gross placental pathology, detection of *Chlamydia abortus* organisms in placental smears and detection of genomic DNA in placental cotyledons of vaccinated ewes that were challenged with *C. abortus* at day 70 of gestation (groups 1–4), of infected control ewes (group 5) and of uninfected control ewes (group 6).

Group	Pregnancy Outcome ^1^	No. Ewes	Lesions ^2^	Smears ^3^	Placental qPCR ^4^	Placental qPCR Load ^5^
1	Lambed	24	24−	24−	24-	1.75 (1.17)
2	Lambed	25	25−	25−	3+, 22−	34.71 (1.19)
3	Lambed	22	22−	22−	3+, 19−	2.80 (1.48)
	Aborted	1	1+	1+	1+	1,711,058 (1.14) ^6^
4	Lambed	22	22−	22−	2+, 20−	5.25 (1.58)
5	Lambed	18	2+, 16−	2+, 16−	17+, 1−	1481 (1.50)
	Aborted	7	7+	7+	7+	8,222,991 (1.86)
6	Lambed	23	23−	23−	23−	6.12 (1.27)

Group 1, 2 × 10 μg COMC; group 2, 1 × 20 μg COMC; group 3, 2 × 5 μg COMC; and group 4, 1 × 10 μg COMC. Group 5, challenge controls. Group 6, negative controls. ^1^ See Table 1. ^2^ Number of ewes with gross pathological lesions characteristic of *C. abortus* infection evident in one or more placentas: +, positive; −, negative. ^3^ Number of ewes with chlamydial organisms detected following modified Ziehl–Neelsen (mZN) staining of placental smears: +, positive; −, negative. ^4^ Number of ewes with chlamydial organisms detected by quantitative PCR (qPCR) of the placental smear samples tested by mZN staining: +, positive; −, negative. ^5^ Geometric mean (geometric SEM) of the number of *C. abortus* genomes per 2 μL total extracted DNA detected by qPCR in the placental cotyledons tested by mZN staining. ^6^ Geometric mean (geometric SEM) of two placentas from a single ewe that aborted.

## Data Availability

Data are contained within the article or supplementary material. The original contributions presented in the study are included in the article/supplementary material, and further inquiries can be directed to the corresponding author/s.

## Supplementary Materials

The following supporting information can be downloaded at: https://www.mdpi.com/article/10.3390/ani14203004/s1, Table S1: Raw data on clinical outcome, gestational length, placental gross pathology, mZN and PCR analysis for each experimental group.
