# Supplementary material for: Evaluation of the Protective Efficacy of Different Doses of a Chlamydia abortus Subcellular Vaccine in a Pregnant Sheep Challenge Model for Ovine Enzootic Abortion
Source: Animals (Basel). 2024 Oct 17;14(20):3004. doi: 10.3390/ani14203004 (PMC11504442; doi:10.3390/ani14203004)
Supplement: Supplementary file 1 [file animals-14-03004-s001.zip › animals-3207721-supplementary.pdf]

Group 1: 2 x 10 µg COMC

Ewe No	Lambed (No)	Aborted (No)	Lambed (L)/ Aborted (A)	Length of gestation	Macroscopic examination			Placental smear (mZN)			Placental load (qPCR)			
					P1	P2	P3	P1	P2	P3	P1	P2	P3	
156	1	0	L	149	0			-			0			
159	2	0	L	146	0	0		-	-		0	68.9		
189	2	0	L	144	0	0		-	-		0	0		
220	2	0	L	139	0	0		-	-		6.8	3.3		
228	2	0	L	145	0	0		-	-		0	3.1		
236	2	0	L	146	0	0		-	-		0	0		
431	2	0	L	146	0	0		-	-		0	0		Asphyxiation
938	2	0	L	142	0	0		-	-		0	0		
962	2	0	L	150	0	0		-	-		9.6	0		
986	1	0	L	144	0			-			0			
1009	2	0	L	146	0	0		-	+/-		0	0		
1019	3	0	L	141	0	0	0	-	+/-	-	13.6	3.7	0	
1075	3	0	L	143	0	0	0	-	-	-	5.7	3	0	
1085	3	0	L	145	0	0	0	-	-	-	0	0	0	
1591	1	0	L	146	0			-			0			
1623	2	0	L	144	0	0		-	-		5.9	0		
1712	1	0	L	146	NF			NF			NA			
1880	1	0	L	142	0			-			0			
1903	2	0	L	143	0	0		-	-		5.8	0		
1910	2	0	L	142	0	NF		-	NF		0	NA		
1940	1	0	L	143	0			-			0			
1997	1	0	L	147	0			-			0			
2002	2	0	L	144	NF	0		NF	-		NA	0		
2024	2	0	L	146	0	0		-	-		0	0		
	43	0	24	L										
			0	A										

NF, not found; NA, not applicable

Group 2: 20 µg COMC

Ewe No	Lambed (No)	Aborted (No)	Lambed (L)/ Aborted (A)	Length of gestation	Macroscopic examination		Placental smear (mZN)		Placental load (qPCR)	
					P1	P2	P1	P2	P1	P2
167	2	0	L	147	0	0	-	-	22.5	22.6
172	1	0	L	143	0?		-		0	
194	2	0	L	145	0	0	-	-	12.3	10.6
416	2	0	L	142	0	0	-	-	8.2	69.2
433	2	0	L	143	0	0	-	-	110	88.5
435	2	0	L	145	0	NF	-	NF	66.6	NA
937	1	0	L	146	0		-		31.7	
976	1	0	L	140	0		-		99.6	
992	1	0	L	145	0		-		7.5	
1022	1	0	L	142	NF		NF		NA	
1032	2	0	L	144	0	0	-	-	11.7	30.5
1048	1	0	L	142	0		-		48.9	
1081	2	0	L	145	0	0	-	-	88.7	41.4
1087	2	0	L	140	0	0	-	-	72.5	10
1624	1	0	L	144	0		-		21.2	
1633	1	0	L	141	0		-		172	
1640	2	0	L	143	0	0	-	-	98.6	108.9
1770	1	0	L	146	0		-		31.2	
1905	2	0	L	145	0	0	-	-	35.2	29
1925	2	0	L	143	0	NF	-	NF	14.2	NA
2006	2	0	L	146	0	0	-	-	45.9	102.3
2012	2	0	L	142	0	0	-	-	12.3	11.8
2044	1	0	L	143	0		+/-		31.1	
2049	2	0	L	147	0	0	-	-	96.9	46.3
2060	1	0	L	147	0		-		163.7	
	39	0	25	L						
			0	A						

NF, not found; NA, not applicable

Group 3: 2 x 5 µg COMC

Ewe No	Lambed (No)	Aborted (No)	Lambed (L)/ Aborted (A)	Length of gestation	Macroscopic examination			Placental smear (mZN)			Placental load (qPCR)		
					P1	P2	P3	P1	P2	P3	P1	P2	P3
154	2	0	L	147	0	0		-	-		0	0	
173	1	0	L	151	0			-			0		
185	1	0	L	150	0			-			0		
199	1	0	L	150	0						12200		
207	2	0	L	146	0	0		-	-		0	0	
423	1	0	L	142	0			-			16.8		
921	2	0	L	143	0	NF		-	NF		0	NA	
940	2	0	L	145	0	0		-	-		0	0	
993	2	0	L	146	0	0		-	-		4.9	0	
1003	2	0	L	141	0	0		-	-		0	0	
1035	2	0	L	146	0	0		-	-		8.7	0	
1045	2	0	L	147	0	0		-	-		0	0	
1054	1	0	L	147	0			-			0		
1058	3	0	L	146	0	0	0	-	-	-	9.2	0	0
1666	1	0	L	147	0			-			1771.7		
1708	2	0	L	145	0	0		-	-		107.1	23.4	
1791	1	0	L	149	0			-			0		
1914	2	0	L	142	0	NF		-	NF		0	NA	
2011	2	0	L	140	0	0		-	-		0	0	
2040	1	0	L	145	NF			NF			NA		
2055	0	2	A	132	100	100		++	++		1497400	1955200	
2094	2	0	L	147	0	0		-	-		0	0	
2108	1	0	L	140	0			-			0		
	36	2	22 L										
			1 A										

Group 4: 10 µg COMC

Ewe No	Lambed (No)	Aborted (No)	Lambed (L)/ Aborted (A)	Length of gestation	Macroscopic examination		Placental smear (mZN)		Placental load (qPCR)	
					P1	P2	P1	P2	P1	P2
142	2	0	L	145	0	0	-	-	11	12.7
179	1	0	L	141	NF		NF		NA	
205	2	0	L	143	0	NF	NF	-	0	NA
212	1	0	L	145	0		-		659.9	
415	1	0	L	148	0		-		0	
923	1	0	L	145	0		-		0	
941	2	0	L	145	0	0	-	-	32.6	0
965	2	0	L	148	0	0	-	-	0	0
997	1	0	L	146	0		-		0	
1037	2	0	L	146	0	0	-	-	0	0
1049	1	0	L	143	0		-		0	
1063	1	0	L	142	0		+/-		183	
1670	2	0	L	145	0	0	-	-	18.7	0
1828	1	0	L	145	0		-		0	
1891	1	0	L	141	0		-		0	
1927	2	0	L	145	0	0	-	-	95	0
1931	1	0	L	149	0		-		23.5	
2058	1	0	L	147			-		0	
2076	2	0	L	145	0	0	-	-	12.5	0
2097	1	0	L	146	0		-		11.5	
2098	2	0	L	143	0	0	-	-	6.1	0
2155	2	0	L	143	0	NF	NF	-	0	N/A
	32	0	22 L							
			0 A							

Group 5: Challenge Control

Ewe No	Lambed (No)	Aborted (No)	Lambed (L)/ Aborted (A)	Length of gestation	Macroscopic examination			Placental smear (mZN)			Placental load (qPCR)			
					P1	P2	P3	P1	P2	P3	P1	P2	P3	
184	2	0	L	149	0	0		-	-		1156.4	9080		
213	2	0	L	141	0	0		-	-		2420.8	2207		
234	1	0	L	146	0			-			2140.7			
419	0	2	A	136	100	NF		+++	NF		6983300	NA		Weak
933	0	2	A	135	40	95		+++	+++		4536100	1.4E+07		Weak
942	1	1	A	138	5	50		++	+++		93506000	1.4E+07		Weak
954	1	0	L	145	0			-			2659.7			
989	2	0	L	143	0	0		-	-		1916.9	1277		
1010	1	1	A	141	0	50		+	++/+++		22284	2847600		Weak
1020	3	0	L	144	0	0	NF	-	-	NF	569.6	1497	NA	
1059	2	0	L	145	0	0		-	-		3347	3293.4		
1065	1	0	L	146	0			-			1232.6			
1071	2	0	L	143	0	0		-	-		470.3	1137.8		
1682	2	0	L	148	0	NF		+/-	NF		595.3	NF		
1731	0	2	A	121	75	90		+++	++++		15066000	3.7E+07		
1860	2	0	L	143	0	NF		-	NF		1440.8	NF		
1899	1	0	L	139	0			-			1683.4			
1928	1	0	L	142	40			++			6764200			
2014	0	2	A	132	80	100		+++	++		72344000	4534800		Weak
2061	3	0	L	145	5	10	NF	-	-	NF	35.5	30.8	NA	
2087	2	0	L	146	0	0?		-	-		4527.3	1027.4		
2089	1	0	L	133	0			-			1295.4			
2112	1	0	L	145	0			-			1334.3			
2114	2	0	L	144	0	0		-	-		309.1	288.6		
2164	0	1	A	137	25			+++			13376000			
	33	11	18 L											
			7 A											

NF, not found; NA, not applicable

Group 6: Negative Control

Ewe No	Lambed (No)	Aborted (No)	Lambed (L)/ Aborted (A)	Length of gestation	Macroscopic examination P1	Macroscopic examination P2	Macroscopic examination P3	Placental smear (mZN) P1	Placental smear (mZN) P2	Placental smear (mZN) P3	Placental load (qPCR) P1	Placental load (qPCR) P2	Placental load (qPCR) P3
150	1	0	L	147	0			+/-			10.2		
190	2	0	L	148	0	0		-	-		0	16.7	
219	2	0	L	145	0	0		-	-		0	9.7	
430	1	0	L	143	0			-			9.5		
935	2	0	L	139	0	0		-	-		8.1	33.9	
967	1	0	L	149	0			-			0		
1000	2	0	L	147	0	NF		-	NF		9.9	NA	
1021	1	0	L	143	0			-			10		
1041	2	0	L	146	0	NF		-	NF		11.7	NA	
1072	2	0	L	142	0	0		-	-		11.3	0	
1074	2	0	L	146	0	0		-	-		5.8	17.2	
1082	3	0	L	145	0	0	0	-	-	-	0	0	27.7
1592	1	0	L	145	0			-			0		
1754	1	0	L	146	0			-			16		
1844	2	0	L	144	0	0		-	-		0	0	
1897	1	0	L	147	0			-			16.9		
1970	2	0	L	146	0	0		-	-		32.5	5.6	
2030	2	0	L	141	0	0		-	-		52.7	44	
2115	1	0	L	146	0			-			12		
2122	1	0	L	145	0			-			10.7		
2142	2	0	L	144	0	0		-	-		0	0	
2143	2	0	L	146	0	0		-	-		15.8	16.2	
2169	1	0	L	148	0			-			0		
	37	0	23	L									
			0	A									

NF, not found; NA, not applicable
